# Discovery and validation of plasma proteomic biomarkers relating to brain amyloid burden by SOMAscan assay

**DOI:** 10.1016/j.jalz.2019.06.4951

**Published:** 2019-11

**Authors:** Liu Shi, Sarah Westwood, Alison L. Baird, Laura Winchester, Valerija Dobricic, Fabian Kilpert, Shengjun Hong, Andre Franke, Abdul Hye, Nicholas J. Ashton, Angharad R. Morgan, Isabelle Bos, Stephanie J.B. Vos, Noel J. Buckley, Mara ten Kate, Philip Scheltens, Rik Vandenberghe, Silvy Gabel, Karen Meersmans, Sebastiaan Engelborghs, Ellen E. De Roeck, Kristel Sleegers, Giovanni B. Frisoni, Olivier Blin, Jill C. Richardson, Régis Bordet, José L. Molinuevo, Lorena Rami, Anders Wallin, Petronella Kettunen, Magda Tsolaki, Frans Verhey, Alberto Lleó, Daniel Alcolea, Julius Popp, Gwendoline Peyratout, Pablo Martinez-Lage, Mikel Tainta, Peter Johannsen, Charlotte E. Teunissen, Yvonne Freund-Levi, Lutz Frölich, Cristina Legido-Quigley, Frederik Barkhof, Kaj Blennow, Henrik Zetterberg, Susan Baker, B. Paul Morgan, Johannes Streffer, Pieter Jelle Visser, Lars Bertram, Simon Lovestone, Alejo J. Nevado-Holgado

**Affiliations:** a Department of Psychiatry, University of Oxford, Oxford, UK; b Lübeck Interdisciplinary Platform for Genome Analytics, Institutes of Neurogenetics and Cardiogenetics, University of Lübeck, Lübeck, Germany; c Institute of Clinical Molecular Biology, Christian-Albrechts-University of Kiel, Kiel, Germany; d Maurice Wohl Clinical Neuroscience, Institute of Psychiatry, Psychology and Neuroscience, Kings College London, London, UK; e Department of Psychiatry and Neurochemistry, University of Gothenburg, Mölndal, Sweden; f Wallenberg Centre for Molecular & Translational Medicine, University of Gothenburg, Gothenburg, Sweden; g NIHR Biomedical Research Centre for Mental Health and Biomedical Research Unit for Dementia at South London and Maudsley NHS Foundation, London, UK; h Dementia Research Institute Cardiff, Cardiff University, Cardiff, UK; i Department of Psychiatry and Neuropsychology, School for Mental Health and Neuroscience, Alzheimer Centrum Limburg, Maastricht University, Maastricht, the Netherlands; j Alzheimer Center, VU University Medical Center, Amsterdam, the Netherlands; k University Hospital Leuven, Leuven, Belgium; l Laboratory for Cognitive Neurology, Department of Neurosciences, KU Leuven, Belgium; m Center for Neurosciences, Vrije Universiteit Brussel (VUB), Brussels, Belgium; n Reference Center for Biological Markers of Dementia (BIODEM), University of Antwerp, Antwerp, Belgium; o Institute Born-Bunge, University of Antwerp, Antwerp, Belgium; p Department of Neurology, VUB University Hospital Brussels (UZ Brussel), Brussels, Belgium; q Neurodegenerative Brain Diseases Group, Center for Molecular Neurology, VIB, Edegem, Belgium; r University of Geneva, Geneva, Switzerland; s IRCCS Istituto Centro San Giovanni di Dio Fatebenefratelli, Brescia, Italy; t AIX Marseille University, INS, Ap-hm, Marseille, France; u Neurosciences Therapeutic Area, GlaxoSmithKline R&D, Stevenage, UK; v University of Lille, Inserm, CHU Lille, Lille, France; w Alzheimer's disease & other cognitive disorders unit, Hopsital Clínic-IDIBAPS, Barcelona, Spain; x Barcelona Beta Brain Research Center, Universitat Pompeu Fabra, Barcelona, Spain; y Institute of Neuroscience and Physiology, Sahlgrenska Academy at University of Gothenburg, Gothenburg, Sweden; z Nuffield Department of Clinical Neurosciences, University of Oxford, Oxford, UK; aa 1st Department of Neurology, AHEPA University Hospital, Makedonia, Thessaloniki, Greece; bb Hospital de la Santa Creu i Sant Pau, Barcelona, Spain; cc Department of Neurology, Hospital de la Santa Creu i Sant Pau, Barcelona, Spain; dd University Hospital of Lausanne, Lausanne, Switzerland; ee Geriatric Psychiatry, Department of Mental Health and Psychiatry, Geneva University Hospitals, Geneva, Switzerland; ff CITA-Alzheimer Foundation, San Sebastian, Spain; gg Danish Dementia Research Centre, Rigshospitalet, Copenhagen University Hospital, Copenhagen, Denmark; hh Neurochemistry Laboratory, Department of Clinical Chemistry, Amsterdam Neuroscience, Amsterdam University Medical Centers, Vrije Universiteit, Amsterdam, The Netherlands; ii Department of Neurobiology, Caring Sciences and Society (NVS), Division of Clinical Geriatrics, Karolinska Institutet, and Department of Geriatric Medicine, Karolinska University Hospital Huddinge, Stockholm, Sweden; jj Department of Psychiatry in Region Örebro County and School of Medical Sciences, Faculty of Medicine and Health, Örebro University, Örebro, Sweden; kk Department of Old Age Psychiatry, Psychology and Neuroscience, Kings College London, London, UK; ll Department of Geriatric Psychiatry, Zentralinstitut für Seelische Gesundheit, University of Heidelberg, Mannheim, Germany; mm Kings College London, London, UK; nn The Systems Medicine Group, Steno Diabetes Center, Gentofte, Denmark; oo Department of Radiology and Nuclear Medicine, VU University Medical Center, Amsterdam, The Netherland; pp UCL Institutes of Neurology and Healthcare Engineering, London, UK; qq Clinical Neurochemistry Laboratory, Sahlgrenska University Hospital, Mölndal, Sweden; rr UK Dementia Research Institute at UCL, London, UK; ss Department of Neurodegenerative Disease, UCL Institute of Neurology, London, UK; tt Janssen R&D, Titusville, NJ, USA; uu Janssen R&D, LLC, Beerse, Belgium; vv Department of Neurobiology, Care Sciences and Society, Division of Neurogeriatrics, Karolinska Institutet, Stockholm, Sweden; ww Department of Psychology, University of Oslo, Oslo, Norway; xx Janssen-Cilag UK, formerly Department of Psychiatry, University of Oxford, Oxford, UK

**Keywords:** Amyloid β, SOMAscan assay, Plasma proteomics, Replication, Causal relationship, Tau

## Abstract

**Introduction:**

Plasma proteins have been widely studied as candidate biomarkers to predict brain amyloid deposition to increase recruitment efficiency in secondary prevention clinical trials for Alzheimer's disease. Most such biomarker studies are targeted to specific proteins or are biased toward high abundant proteins.

**Methods:**

4001 plasma proteins were measured in two groups of participants (discovery group = 516, replication group = 365) selected from the European Medical Information Framework for Alzheimer's disease Multimodal Biomarker Discovery study, all of whom had measures of amyloid.

**Results:**

A panel of proteins (n = 44), along with age and apolipoprotein E (*APOE*) ε4, predicted brain amyloid deposition with good performance in both the discovery group (area under the curve = 0.78) and the replication group (area under the curve = 0.68). Furthermore, a causal relationship between amyloid and tau was confirmed by Mendelian randomization.

**Discussion:**

The results suggest that high-dimensional plasma protein testing could be a useful and reproducible approach for measuring brain amyloid deposition.

## 1 Introduction

Antiamyloid clinical trials for Alzheimer's disease (AD) have been unsuccessful to date [1]. The failure of such trials was partially caused by the fact that participants enrolled in such trials present clinically with AD but lack amyloidosis [2]. Furthermore, most such trials have been conducted relatively late in the disease process and targeting treatment to earlier presymptomatic or prodromal stages of the disease might have more success [3]. As a consequence, it has increasingly common to conduct clinical trials in preclinical or prodromal disease where AD pathology is confirmed using biomarkers [2], [4]. Currently, the best characterized methods for measuring amyloid pathology are positron emission tomography (PET) imaging measures of brain amyloid deposition and cerebrospinal fluid (CSF) measurement of amyloid β (Aβ) levels [5]. However, these measures are challenging because of invasiveness, cost, and restricted availability [6], [7].

Blood-based biomarkers have therefore been investigated as a less-invasive and potentially cost-effective option for early detection and monitoring of AD pathology. An increasing number of studies [8], [9], [10], [11], [12], including those by ourselves [13], [14], [15], [16], [17], [18], [19], have found that a range of proteins in plasma can reflect neocortical Aβ burden and hence might act as biomarkers. Recent studies have added to these by demonstrating that measures of Aβ in blood can predict brain amyloid with high accuracy [20], [21], [22], further indicating that blood proteins can be effective biomarkers for predicting brain pathology.

The failure of antiamyloid clinical trials also provoked some to question the amyloid cascade hypothesis [23], [24], [25]. Broadly speaking, the amyloid cascade hypothesis states that over time, an imbalance in Aβ production and/or clearance leads to gradual accumulation and aggregation of the peptide in the brain, initiating a neurodegenerative cascade that involves not only amyloid deposition but also inflammation, tau pathology, neuronal dysfunction, and loss [26], [27]. Despite some reservations regarding this hypothesis prompted by the failure of clinical trials, amyloid still remains a central part of our understanding of the pathophysiology of the disease. Nonetheless, assessing the relationship between amyloid and other AD pathologies has become increasingly important.

With this in mind, we embarked on the study reported here, with two main objectives; first to identify a plasma molecular signature of amyloid pathology for potential use as a biomarker for preclinical or prodromal detection of disease and second to investigate the causal relationship between amyloid and other AD pathologies. We report here a replicating signature of disease reflecting brain amyloid load and other features of AD, including tau pathology, in blood. Then using Mendelian randomization (MR) [28], an approach more often used in genetic studies, we find a causal relationship between amyloid and tau while such reverse relationship was not found.

## 2 Methods

### 2.1 Participants: EMIF-AD Multimodal Biomarker Discovery study

The EMIF-AD Multimodal Biomarker Discovery (MBD) study is part of the European Medical Information Framework for Alzheimer's disease (EMIF-AD; http://www.emif.eu); a public-private precompetitive cross European program seeking to make data, and in these case samples, reuseable for secondary studies of neurodegeneration. The design of the EMIF-AD MBD study has been described previously [29]. Briefly, 1221 participants meeting specified and previously reported inclusion criteria were identified from 11 preexisting European cohorts (“parent cohorts”) using the EMIF catalog (https://emif-catalogue.eu) and associated tools. Each parent cohort was approved by local medical ethics committees. As reported previously [29], a critical criterion for inclusion was that a measure of brain amyloid load was available, using either CSF Aβ or amyloid PET scan. The classification of amyloid burden (high/low) in the EMIF-AD MBD cohort has been described previously [29]. Furthermore, Aβ measurements were combined into a continuous variable using z-scoring, using the mean and standard deviation of the control subjects as a reference [29].

In addition to the primary outcome variable of amyloid load, another five markers in CSF were measured including total tau (t-tau), phosphorylated tau (p-tau), neurofilament light chain (NFL), neurogranin, and YKL-40 [29]. The levels of t-tau and p-tau in CSF were measured locally, and the local cutoff point was used to determine their status (high/low). Consequently, the p-tau and t-tau values were Z-scored with controls within each data set as a reference. The other three CSF markers (NFL, neurogranin, and YKL-40) were measured in a central laboratory (Gothenburg University, Sweden), and their status (high/low) was determined by the median value of each marker within the whole data set.

In addition, the following AD-related phenotypes were also measured: magnetic resonance imaging values of hippocampal volume; clinical assessments including baseline diagnosis, baseline Mini–Mental State Examination (MMSE) score, and mild cognitive impairment (MCI) conversion; and finally, apolipoprotein E (*APOE)* and genomewide single nucleotide polymorphism (SNP) genotyping [29]. SNP assays were conducted using the “Global Screening Array” (Illumina, Inc.) using standard procedures from 250 ng of DNA extracted from whole blood [30]. Raw data processing and initial quality control (QC) were performed in GenomeStudio (v2.0.2) using GenTrain (v3.0) as clustering algorithm generating genotypes for 645,896 SNPs. Postprocessing was performed in PLINK (v1.9) using standard QC thresholds (i.e., sample genotyping efficiency > 95%, SNP genotyping efficiency > 98%, departures from Hardy Weinberg equilibrium at *P* value < .000005, and a minor allele frequency [MAF] of < 0.01). Eventually, this left 490,717 SNPs for analysis. Genotypes for reconstruction of the *APOE* ε2/3/4 haplotypes were either generated on the Global Screening Array (rs7412) or by manual genotyping (rs429358) as described previously [31]. We classified individuals as *APOE* ε4 carriers or noncarriers according to their genotype status at rs429358 (C-allele = ε4).

The 1221 subjects selected for the EMIF-AD MBD cohort included 492 healthy controls (HCs), 527 MCI, and 202 AD participants. Among these participants, we included in the study all those that had plasma samples available (total 881). These samples were then divided into two separated groups, with each group of samples being processed independently. Group 1 was used as a discovery group and included 150 HCs (29.1%), 188 MCI (36.4%), and 178 AD (34.5%). Group 2 was used for validation and included 161 HCs (44.1%), 198 MCI (54.2%), and 6 AD (1.6%). Furthermore, we sought to maintain amyloid balance in controls and MCI individuals given that the objective is to identify biomarkers of amyloid pathology in preclinical stage. As a result, in this combined group of samples from people in the HC and MCI categories (total n = 697), there were 338 with and 359 without amyloid pathology.

### 2.2 SOMAscan assay

We measured proteins in plasma using the SOMAscan assay platform (SomaLogic Inc.). SOMAscan is an aptamer-based assay allowing for the simultaneous measurement and quantification of, in the version used here, 4001 proteins. The assay uses chemically modified nucleotides to transform a protein signal into a nucleotide signal that can be quantified using relative fluorescence on microarrays [32]. As noted previously, groups of samples were measured independently. Forty subjects were tested in both batches to normalize the data across assay runs.

### 2.3 Statistical analysis

All statistical analyses were completed using R (version 3.3.2). The Mann-Whitney U test was used to compare participants' characteristics between high- and low-amyloid categories in both groups. The association between proteins and amyloid Z-score was calculated by partial Spearman correlation, adjusting for five covariates including age, sex, *APOE* ε4 status, study of origin, and number of blood freeze-thaw cycles. False discovery rate correction was used to correct *P* values for multiple comparisons. Proteins that were differentially expressed at a significance level of *P* < .05 were further nominated for pathway analysis using the Metascape software (http://metascape.org/gp/index.html#/main/step1). Briefly, differentially expressed proteins were inputted as “protein list” and all 4001 proteins measured by SOMAscan assay were used as “background.” This enrichment analysis was performed on the KEGG database.

### 2.4 Machine learning

Machine learning was used to identify optimal multivariate signatures, including both proteins and demographic data (age and *APOE* ε4) as input features, to differentiate between high and low Aβ status in group 1. First, the effects of study and blood freeze-thaw cycle on proteins were removed by linear regression and the residuals were used for analysis. Subsequently, Lasso was used to select the “n” top input features that best differentiated high from low Aβ. Support vector machine classifiers were then built using from 1 to 100 features to predict the outcome under 10-fold cross-validation. Those features that produced best performance were selected from group 1. To verify replication, the optimal classifier was then validated in the second and combined cohorts for distinguishing Aβ status and other AD endophenotypes.

### 2.5 Mendelian randomization

MR was used to investigate the causal relationship between amyloid and tau pathology. MR uses genetic variants as an instrument when they are robustly associated with a risk factor (exposure), with the fundamental assumption that they are associated with the studied outcome through that exposure. As genetic variants cannot be altered and are static throughout an individual's lifetime, reverse causation can be excluded, rendering MR a powerful tool to examine causality between the exposure and outcome [28].

To explore the causal relationship between amyloid and tau (both t-tau and p-tau), we first performed a systematic review of the literature to search SNPs that were significantly associated with amyloid but not with tau or p-tau. Overall, 78 SNPs were found to be significantly associated with abnormal amyloid pathology measured by PET scan, CSF amyloid, or postmortem measurement (Supplementary Table 1). Six SNPs were excluded including 3 which were also associated with t-tau and p-tau and 3 which were within 1 Mb of *APOE* (i.e., *APOC1* and *TOMM40*), given that *APOE* is strongly associated with both amyloid and tau. Of the remaining SNPs, 6 were not measured using the assay we used, leaving 66 SNPs for further analysis (Supplementary Table 1). The association of these 66 SNPs with the Z-score of amyloid, t-tau, and p-tau was calculated using PLINK (v1.7). Finally, based on the association, we used four MR approaches, including MR Egger linear regression, weighted median, inverse-variance weighted (IVW), and simple median [33], to investigate the causal relationship between amyloid and tau (both t-tau and p-tau). Each MR method is based on different assumptions. The Egger method is sensitive to SNP pleiotropy and allows the estimation of underlying bias by allowing a non-zero estimate for the intercept of the calculated ratio of β values [34]. The weighted median method uses a median of the individual causal estimate per SNP, which is calculated from the ratio estimates of outcome's regression coefficient divided by exposure [35]. The IVW method uses the same ratio estimates but incorporates IVWs into the final summary estimate [36]. Comparing estimates from all the methods shows the robustness of the overall analysis. Furthermore, to determine if there was any single SNP driving the relationship, we performed a leave-one-out analysis where the MR is performed removing a different SNP in each iteration [37].

A similar approach was performed to investigate the causal relationship between t-tau and amyloid. Using the same approach, we identified 21 SNPs that were significantly associated with the abnormal CSF t-tau level but not with amyloid (Supplementary Table 2). After checking the effect allele, eighteen SNPs passed QC and were used for MR analysis (Supplementary Table 2). The causal relationship between p-tau and amyloid was not presented because few SNPs were found to be strongly associated with p-tau. Therefore, this approach was underpowered for making causal inferences.

## 3 Results

### 3.1 Subject demographics

The EMIF-MBD cohort comprises 1221 subjects of which 881 had samples available for this study. As described in the Methods section, these were divided into two groups for discovery and validation purposes. Table 1 shows the characteristics of the participants from two groups split by amyloid status (high/low). For both groups, the participants with high amyloid were older (*P* = .04 in group 1, *P* < .001 in group 2), the MMSE was lower (*P* < .001 in group 1, *P* < .001 in group 2), and the prevalence of *APOE* ε4 carriers was higher (*P* < .001 in group 1, *P* < .001 in group 2). A significant difference in sex was found in group 1 (*P* = .002), whereas there was no difference in group 2 (*P* = .14).

**Table 1:** Demographics of the study population

Characteristics	Group 1 (n = 516)			Group 2 (n = 365)		
	High Aβ (n = 369)	Low Aβ (n = 147)	*P* value	High Aβ (n = 134)	Low Aβ (n = 231)	*P* value
Aβ Z-score	−1.39 ± 0.50	0.49 ± 0.57	<.001∗	−1.22 ± 0.40	0.52 ± 0.67	<.001∗
Age (yrs)	70.0 ± 8.2	68.3 ± 8.4	.04∗	70.0 ± 8.3	64.9 ± 8.1	<.001∗
Female sex, N (%)	210 (57)	62 (42)	.002∗	70 (52)	139 (60)	.14
Male sex, N (%)	159 (43)	85 (58)	.002∗	64 (48)	92 (40)	.14
*APOE* ε4+, N (%)	244 (66)	43 (29)	<.001∗	76 (57)	53 (23)	<.001∗
*APOE* ε4−, N (%)	125 (34)	104 (71)	<.001∗	58 (43)	178 (77)	<.001∗
MMSE	24.1 ± 4.6	27.0 ± 3.2	<.001∗	26.9 ± 2.7	28.2 ± 1.8	<.001∗

NOTE. Percentage of cases is shown in brackets for female and male sex, as well as *APOE* ε4 carriers and noncarriers in each amyloid β (Aβ) category. *P* values compare each demographic across the high Aβ and low Aβ groups. Group 1 containing 516 participants was used for discovery, and group 2 of 365 participants for validation.

Abbreviations: *APOE*, apolipoprotein E; MMSE, Mini–Mental State Examination.

∗ *P* < .05.

### 3.2 Proteins significantly associated with amyloid Z-score in both groups

In this study, we sought to find plasma biomarkers related to amyloid pathology independent of diagnosis and so combined samples from study participants across the AD diagnostic spectrum (HC, MCI, and AD) and used measures of amyloid pathology as the principle outcome measure. Combining samples in this way reflects the operational need for a biomarker predicting brain amyloid pathology. Recent experience suggests that up to 20% of people with a diagnosis of AD in clinical trials had little or no brain amyloid pathology, demonstrating that clinical classification is an inadequate predictor of brain pathology. Moreover, a biomarker of brain pathology in all three states—HC, MCI, and AD—would have value. In HC, a marker of brain amyloid would help to identify preclinical AD, in MCI, to identify prodromal AD, and in AD itself, to add to the accuracy of diagnosis.

**Fig. 1:**
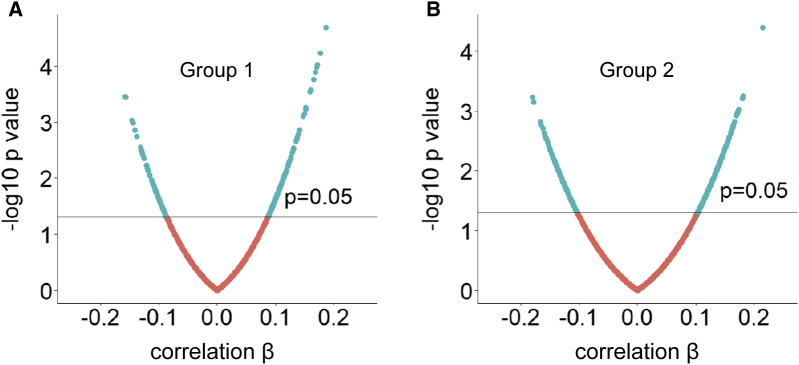
Correlation of plasma proteins with amyloid Z-score by partial Spearman correlation. (A and B) Volcano plot of proteins obtained from group 1 and group 2, respectively.

Overall, 3676 and 3916 proteins passed QC measures in group 1 and group 2, respectively. Using partial correlation to analyze the association between proteins and amyloid Z-score, we found that in group 1, 301 proteins reached statistical significance (*P* < .05) and 5 of them reached false discovery rate (*q* < 0.1) (Supplementary Table 3), corrected for multiple comparisons. In group 2, 536 proteins reached statistical significance (*P* < .05) while none passed false discovery rate. Fig. 1A and B shows volcano plots of proteins from both groups with cutoff *P* = .05.

We then merged the lists of differentially expressed proteins obtained from both groups finding a total of 786 proteins associated with high versus low amyloid. This number of dysregulated proteins was significantly higher than that obtained by chance alone (*P* < .001). We then used the Metascape software, performed on the KEGG database, to assess the biological significance of these dysregulated proteins. Pathway analysis of the 786 differentially expressed proteins revealed 10 significantly enriched pathways: arginine biosynthesis (*P* = .003), inflammatory mediator regulation of TRP channels (*P* = .007), glycerophospholipid metabolism, (*P* = .010), citrate cycle (*P* = .012), Notch signaling (*P* = .016), RNA degradation (*P* = .023), type II diabetes mellitus (*P* = .038), peroxisome (*P* = .038), fatty acid degradation (*P* = .041), and NOD-like receptor signaling (*P* = .043) pathways.

### 3.3 Multiprotein classifier of amyloid normal/abnormal status and other AD endophenotypes

**Fig. 2:**
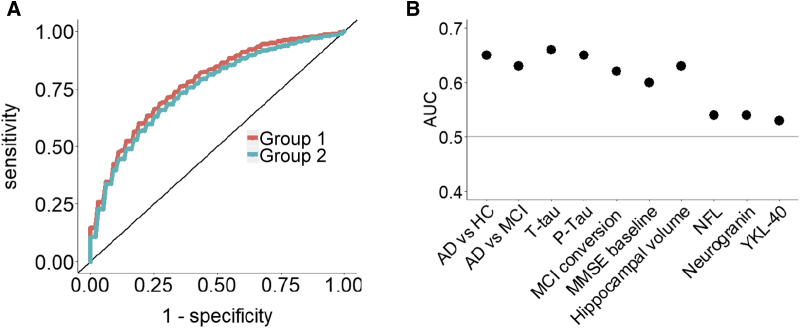
(A) AUC of the 46 signatures to differentiate amyloid status in group 1 and group 2. (B) AUC of the 46 signatures to differentiate other AD endophenotypes in the combined cohort. Abbreviations: AUC, area under the curve; HC, healthy control; MCI, mild cognitive impairment; t-tau, total tau; p-tau, phosphorylated tau; MMSE, Mini–Mental State Examination; NFL, neurofilament light chain.

Having demonstrated that plasma proteins are significantly altered in samples from people with abnormal Aβ levels, we then sought to find a minimal signal from these plasma data that might act as a biomarker indicative of brain amyloid pathology. To do this we performed machine learning with 10-fold cross-validation in group 1 to identify the optimal multivariate signatures that differentiated between high- and low-Aβ individuals. We first used demographic variables (age, sex, and education) with *APOE* ε4 as input features to predict amyloid burden. Results showed that a combination of age and *APOE* ε4 achieved the highest predictive value AUC of 0.70. Then, we added all measured proteins to these demographic variables and we found that a panel of 46 features achieved the highest predictive value AUC of 0.78. The input features automatically selected by the classifier were age, *APOE* ε4, and 44 proteins (Supplementary Table 4). The AUC in training and testing sets for different number of input features is shown in Supplementary Fig. 1. We then investigated the performance of this classifier on differentiating amyloid status in the second and independent group, maintaining the parameters obtained from group 1. The results showed that the same panel achieved an AUC of 0.68, demonstrating good replication (Fig. 2A).

We further investigated the performance of the same panel to predict other AD endophenotypes in the combined cohort (combining groups one and two), including discriminating AD from HC and AD from MCI, differentiating status of t-tau, p-tau, MCI conversion, baseline MMSE score, hippocampal volume, and other three CSF biomarkers including NFL, neurogranin, and YKL-40. As shown in Fig. 2B, the panel predicted most of these AD phenotypes with an AUC of >0.6, except for CSF NFL, neurogranin, and YKL-40. The sample size and AUC of each AD phenotype is shown in Supplementary Table 5.

### 3.4 Causal relationship between amyloid and tau pathology

We then sought to use these data to address the critically important question of causality in relation to AD pathological features. To investigate the causal relationship between amyloid and tau, we first identified SNPs associated with amyloid pathology from systematic review of the literature. We found 72 SNPs (Supplementary Table 1) significantly associated with abnormal amyloid but not with tau pathology of which 66 were assayed using the array used in this study (Supplementary Table 1) and hence available for MR analysis. Results showed that amyloid was associated with a moderate alteration in t-tau when using three MR approaches including the Egger method (β = 0.85, se = 0.15, 95% CI [0.54, 1.15], *P* < .001), the IVW method (β = 0.86, standard error of the effect size [se] = 0.11, 95% CI [0.65, 1.08], *P* < .001), and weighted median method (β = 0.73, se = 0.22, 95% CI [0.31, 1.16], *P* < .001), though not from simple median method (β = 0.81, se = 0.50, 95% CI [-0.17, 1.80], *P* = .11; Table 2, Supplementary Fig. 2). Altered amyloid was also associated with p-tau in all four MR approaches (Table 2, Supplementary Fig. 2). Results from leave-one-out analysis demonstrated that no single SNP was driving the majority of the association signal between amyloid and either t-tau or p-tau (Supplementary Fig. 3). In addition, we explored the causal relationship between amyloid and other three CSF markers including NFL, neurogranin, and YKL-40; no significant association between amyloid and these three markers was found (Supplementary Table 6).

**Table 2:** MR estimates of the causal effect of amyloid on t-tau and p-tau as well as the causal effect of t-tau on amyloid

Study type	Methods	β	se	95% CI	*P* value
Amyloid SNPs with t-tau	MR Egger	0.85	0.15	0.54 to 1.15	<.001
	Weighted median	0.73	0.22	0.31 to 1.16	<.001
	IVW	0.86	0.11	0.65 to 1.08	<.001
	Simple median	0.81	0.50	−0.17 to 1.80	.110
Amyloid SNPs with p-tau	MR Egger	0.73	0.17	0.39 to 1.06	<.001
	Weighted median	0.62	0.23	0.17 to 1.06	.006
	IVW	0.86	0.12	0.62 to 1.10	<.001
	Simple median	1.31	0.52	0.29 to 2.33	.014
t-tau SNPs with amyloid	MR Egger	−0.45	0.36	−1.15 to 0.26	.23
	Weighted median	−0.31	0.29	−0.87 to 0.25	.27
	IVW	−0.02	0.20	−0.42 to 0.38	.93
	Simple median	−0.20	2.05	−4.22 to 3.81	.92

Abbreviations: β, β coefficient; CI, confidence interval; IVW, inverse-variance weighted; MR, Mendelian randomization; t-tau, total tau; p-tau, phosphorylated tau; SNP, single nucleotide polymorphism.

We then reversed the direction of analysis and investigated whether there was a causal relationship between t-tau and amyloid. Twenty-one SNPs (Supplementary Table 2) were found to be significantly associated with the abnormal CSF t-tau level from systematic review, among which eighteen SNPs (Supplementary Table 2) passed QC and were used for MR analysis. Results showed no causal relationship between t-tau and amyloid from any of the four MR approaches (Table 2). We also investigated the causal relationship between t-tau and other three CSF markers (NFL, neurogranin, and YKL-40). No causal relationship between t-tau and NFL was found, whereas such relationship was found between t-tau and neurogranin as well as between t-tau and YKL-40 (Supplementary Table 6).

## 4 Discussion

In this study, we used the SOMAscan assay to measure 4001 proteins in plasma in two groups of subjects from the EMIF-AD MBD study. Using machine learning, we identified a panel of 44 proteins that, along with age and *APOE* ε4, predicted measures of central amyloid with good performance in both the discovery group (AUC = 0.78) and the replication group (AUC = 0.68). The same panel also predicted other AD phenotypes, including CSF t-tau, CSF p-tau, MCI conversion, baseline MMSE score, and hippocampal volume. Moreover, a causal relationship between amyloid and t-tau and with p-tau was confirmed using a MR approach, whereas evidence for the reverse relationship between t-tau and amyloid was not found.

These results are in concordance with one of our previous studies [18], in which we used the same SOMAscan assay to measure plasma proteins in 58 cognitively healthy men. In this previous study, 667 proteins were significantly associated with CSF amyloid measurement, and of these 167 proteins (25%) overlapped with the differentially expressed proteins obtained in the present study. Furthermore, the number of overlapping proteins was significantly higher than expected from chance alone (*P* = .01), confirming that the SOMAscan assay may be a useful and powerful screening tool for identifying plasma proteins reflecting amyloid deposition in early, even preclinical, phases of disease and across the clinical AD spectrum as in the present study.

Pathway analysis of the 786 differentially expressed proteins revealed 10 significantly enriched pathways. When comparing these enriched pathways with AlzPathway, a comprehensive map of AD signaling and related pathways [38], [39], we found two pathways common to both; Notch signaling and type II diabetes mellitus. This finding is interesting given that multiple lines of evidence have previously shown that Notch signaling and type II diabetes mellitus are associated with AD [40], [41], [42], [43]. Our results add weight to this finding and indicate that proteins involved in these two pathways could be treatment targets of AD.

From the discovery phase of the present study, a panel of 46 features achieved a high AUC in discriminating high from low brain amyloid (Fig. 2A). Age and *APOE* ε4 contributed to the model, as in previous studies seeking biomarkers indicative of brain amyloid; unsurprisingly given that both age and *APOE* genotype are highly associated with amyloid pathology [44], [45], [46], [47], [48]. Despite this, the protein signature consisting of 44 proteins achieved a greater discrimination performance in the discovery group than age and *APOE* ε4 (0.78 vs. 0.70). In this study, as the sample collection was deliberately selected to overrepresent high amyloid individuals, the proportion of *APOE* ε4 carriers in the sample collection is higher than in the general population (47.2% vs. 10-20% [49]). In the real world of clinical trials and indeed clinical practice, *APOE* is of less value in identifying people with likely AD pathology as most people in the early stages of disease are *APOE* ε4 negative. Because of this, an algorithm that relies on *APOE* genotype to identify people for inclusion in clinical trials is of limited value, and hence, the identification of additional markers, such as we report here, is even more valuable than a modest increase in AUC over a predictor that includes *APOE* would suggest.

This study is the largest we are aware of to report a plasma biomarker indicative of AD pathology both in terms of the number of proteins assayed and in samples size. Recently, Nakamura et al. (2018) [20] and Ovod et al. (2017) [21] reported methods to measure plasma Aβ directly, and Ashton et al. (2019) [50] used high-resolution mass spectrometry to identify plasma proteins that correlate with altered central amyloid. In all three cases, these assays achieve higher measures of accuracy than we report. However, our study is considerably larger and conducted in a mixed cohort derived from multiple different sources. By virtue of this size and the likely heterogeneity of the sample characteristics, it is possible that the results we report here are likely to withstand the rigors of application in the real-world situations of multicentre, multinational complex clinical trials. Moreover, the analytical platform we use here is clinic-ready and relatively low cost. Finally, using the panel we describe could have significant economic benefits. Based on the performance of our panel (AUC = 0.78, sensitivity = 0.84, specificity = 0.54), we obtain a likelihood ratio of 1.82, suggesting that the panel could improve approximately 15% in amyloid detection [51] relative to current practice. Given that screen failure rates for amyloid positive recruitment to clinical trials is approximately 70%, then to recruit 100 individuals there is a need to enroll 333 participants with a cost of approximately $1.7 million ($5000 per scan). A 15% improvement as we demonstrate would reduce the number needed to screen from 333 to 222, with a cost saving of $0.55 million per 100 participants of a clinical trial. Therefore, as trials typically seek to recruit large numbers of participants, especially in late phase, we believe that despite the moderate improvement of AUC, these 44 proteins obtained from our large sample size study are strong candidates and worthy of further investigation.

The National Biomarker Development Alliance proposes 6 steps for any biomarker development (nbdabiomarkers.org/) including early discovery, translatable discovery, assay development, assay performance, biomarker quantification, and biomarker validation. Here, we have discovered and replicated 44 nominated biomarkers, the subsequent phase of research will be “translatable discovery,” indicating that we need to replicate these 44 proteins in even larger independent cohorts to further minimize overfitting and to obtain a smaller panel of proteins. One of the advantages of the platform that we used here, SOMAscan, is that it is a high-quality assay platform already in increasingly wide research use and appropriate for clinical implementation. In the short to medium term, the results we present here might be used in prescreening processes to recruit people to clinical trials where screening includes determination of amyloid status by CSF or PET markers. Currently, the very high screen failure rate at this stage slows clinical development and adds substantially to the cost of clinical trials while also exposing large numbers of potential participants to relatively invasive tests. Our data suggest that this prescreen failure when seeking people with brain pathology could be reduced using a simple blood assay. In future years, an assay such as this could be used as part of a clinical workup directing at risk individuals to specific testing—such as by CSF measures or using PET imaging—before therapeutic intervention.

In addition to predicting amyloid status, the 44 proteins along with age and *APOE* ε4 could also predict other AD endophenotypes including discriminating AD from HC and AD from MCI, differentiating status of t-tau, p-tau, MCI conversion, baseline MMSE score, and hippocampal volume. These findings are in concordance with some recent studies [52], [53] and further demonstrate the association of amyloid pathology with other pathologies of AD. Moreover, these findings are potentially useful in clinical trial settings especially as increasing number of clinical trials are directed at tau-related targets [1]. The biomarker panel that we report here could potentially be used in very large numbers of people to identify those likely to have both amyloid and tau pathology, even in preclinical phase.

Despite more than two decades of study, the mechanism of AD pathology is still disputed with serial trials failure provoking some to question the amyloid cascade hypothesis. Broadly speaking, this states that the generation of Aβ leads, through a series of largely unknown steps, to tau pathology and hence neuronal dysfunction and dementia [26], [27]. Using the data generated here we confirmed the relationship between amyloid and tau pathology using two approaches. First, the proteins selected from amyloid prediction also predict tau pathology (CSF t-tau and p-tau). Second, we used MR, a method more commonly used in genetic studies to go beyond association to causality, finding that higher amyloid levels drive a moderate increase in both t-tau and p-tau but that the reverse (t-tau driving amyloid pathology) is not true. Our results confirm the crucial role of amyloid in driving tau pathology marking the onset of preclinical AD, and consistent with previous findings [53], [54], [55].

Interestingly, no such association was found between amyloid and other three CSF biomarkers (NFL, neurogranin, and YKL-40), either from using proteins to discriminate their levels or from MR analysis. This suggests that these three biomarkers (NFL, neurogranin, and YKL-40) play different roles in AD independent of, or at least remote from, amyloid pathology. Although their role is not understood completely, changes in NFL are thought to reflect axonal damage [56], changes in neurogranin to reflect synaptic dysfunction [57], and changes in YKL-40 to provide a measure of neuroinflammation [58], [59]. Our data suggest that amyloid is strongly and causally associated with tau pathology but not with synaptic dysfunction or neuroinflammation. Instead, we found that tau is causally associated with neurogranin and YKL-40, indicating that synaptic dysfunction and neuroinflammation are consequences of tau-pathological processes. Interestingly, similar observations have been made and are in line with our findings. For example, in multiple transgenic models, amyloid pathology results in very little neuronal damage, synaptic loss, or indeed neuroinflammation, and where such pathological consequences do exist, they are abolished in the context of ablation of tau [60], [61], [62]. Although these findings are necessarily preliminary, they suggest that proteomic and perhaps other high-dimensionality molecular biomarker studies might also contribute to an understanding of pathological processes in disease.

We acknowledge that the sample origin is a limitation of this study. Given that both the discovery and replication samples are taken from the same meta-sample collection, it is not an independent validation in the traditional sense. However, because the EMIF-AD MBD study is a multicentre study and collected samples from 11 centers across Europe, the meta-collection reflects the challenges faced in the real world of multisite and multinational clinical studies. Therefore, the replication obtained in this study in such a collection of samples has a higher probability of effective utility in practice.

In conclusion, our results strongly suggest that testing high-dimensional plasma protein levels is a useful and reproducible approach to measure central nervous system amyloid deposition. This can be potentially applied as a prescreen to preselect patients for further selection procedures for clinical trials, thus reducing the cost incurred to clinical trials by screen failure. Furthermore, we confirmed the causal relationship between amyloid and tau pathology from MR.

**Research in Context**

1. Systematic review: We reviewed the literature using PubMed sources, meeting abstracts, and presentations. Plasma proteins have been widely studied as candidate biomarkers to predict brain amyloid deposition, but most such studies are targeted to specific proteins or are biased toward high abundant proteins. Furthermore, the mechanism of AD pathology is still disputed, with serial trials failure provoking some to question the amyloid cascade hypothesis.
2. Interpretation: Our findings showed that testing plasma proteins using the high-dimensionality SOMAscan assay could be a useful and reproducible approach for indicating brain amyloid deposition as well as tau pathology. Furthermore, we confirmed the crucial role of amyloid pathology in AD process with a novel use of Mendelian randomization.
3. Future directions: Although it looks promising that a blood test could be used as a tool to preselect individuals for further clinical trials, the plasma biomarkers identified from this study need to be further validated in a larger and independent cohort.
