# Supplementary material for: Discovery and validation of plasma proteomic biomarkers relating to brain amyloid burden by SOMAscan assay
Source: Alzheimers Dement. 2019 Nov;15(11):1478–88. doi: 10.1016/j.jalz.2019.06.4951 (PMC6880298; doi:10.1016/j.jalz.2019.06.4951)
Supplement: Supplementary Material [file mmc1.docx]

| **Genes** | **SNPs** | **Aβ measurement** | **Reference** |
| --- | --- | --- | --- |
| CLU | rs11136000 | CSF Aβ | Elias-Sonnenschein et al., (2013) [1] |
|  |  | PET | Tan et al., (2016) [2] |
| ABCA7 | rs3752246 | PET | Apostolova et al., (2018) [3] |
|  |  | PET | Hughes et al., (2014) [4] |
| ABCA7 | rs3764650 | PET | Apostolova et al., (2018) [3] |
|  |  | Postmortem NP | Shulman et al., (2013) [5] |
| EPHA1 | rs11771145 | CSF Aβ | Martiskainen et al., (2015) [6] |
|  |  | PET | Apostolova et al., (2018) [3] |
| CALHM1 | rs2986017 | CSF Aβ | Koppel et al., (2011) [7] |
|  |  | CSF Aβ | Kauwe et al., (2010) [8] |
| MS4A4A | rs2304933 | CSF Aβ | Elias-Sonnenschein et al., (2013) [1] |
| CLU | rs1532278 | PET | Tan et al., (2016) [2] |
| CLU | rs2279590 | PET | Tan et al., (2016) [2] |
| CLU | rs7982 | PET | Tan et al., (2016) [2] |
| CLU | rs9331888 | PET | Tan et al., (2016) [2] |
| CR1 | rs4844609 | PET | Zhu et al., (2017) [9] |
| ABCA7 | rs3752242 | PET | Zhao et al., (2016) [10] |
| ABCA7 | rs3752240 | PET | Zhao et al., (2016) [10] |
| ABCA7 | rs4147912 | PET | Zhao et al., (2016) [10] |
| SLC24A4 | rs10498633 | CSF Aβ | Martiskainen et al., (2015) [6] |
| PICALM | rs3851179 | Autopsy Aβ | Kok et al., (2011) [11] |
| PICALM | rs541458 | CSF Aβ | Schjeide et al., (2011) [12] |
| CR1 | rs6656401 | Autopsy Aβ | Chibnik et al., (2011) [13] |
| SORL1 | rs668387 | CSF Aβ | Alexopoulus et al., (2011) [14] |
| CYP19A1 | rs2899472 | CSF Aβ | Han et al., (2010) [15] |
| NCAM2 | rs1022442 | CSF Aβ | Han et al., (2010) [15] |
| ARL5B | rs11015839 | CSF Aβ | Han et al., (2010) [15] |
| ACE | rs1800764 | CSF Aβ | Kauwe et al., (2009) [16] |
| DHCR24 | rs7551288 | PET | Swaminathan et al., (2012) [17] |
| FRA10AC1 | rs116953792 | CSF Aβ | Li et al., (2015) [18] |
| FRA10AC1 | rs10509663 | CSF Aβ | Li et al., (2015) [18] |
| SUCLG2 | rs62256378 | CSF Aβ | Ramirez et al., (2014) [19] |
| GSK3B | rs6775397 | CSF Aβ | Kettunen et al., (2015) [20] |
| NR1H3 | rs7120118 | Autopsy Aβ | Natunen et al., (2013) [21] |
| CD2AP | rs9349407 | Postmortem NP | Shulman et al., (2013) [5] |
| APP | rs2829887 | Postmortem NP | Shulman et al., (2013) [5] |
| CR1 | rs6701713 | Postmortem NP | Shulman et al., (2013) [5] |
| KCNIP4 | rs6817475 | Postmortem NP | Shulman et al., (2013) [5] |
| PTGS1 | rs12551233 | Postmortem NP | Shulman et al., (2013) [5] |
| HLA-DQA2 | rs3892710 | Postmortem NP | Shulman et al., (2013) [5] |
| / | rs9407730 | Postmortem NP | Shulman et al., (2013) [5] |
| / | rs4642480 | Postmortem NP | Shulman et al., (2013) [5] |
| NMNAT3 | rs4564921 | Postmortem NP | Shulman et al., (2013) [5] |
| NPAS3 | rs10149826 | Postmortem NP | Shulman et al., (2013) [5] |
| PARD3B | rs12613305 | Postmortem NP | Shulman et al., (2013) [5] |
| GALNT7 | rs62341097 | Postmortem NP | Beecham et al., (2014) [22] |
| TREM1 | rs6910730 | Postmortem NP | Replogle et al., (2015) [23] |
| ZCWPW1 | rs1476679 | PET | Apostolova et al., (2018) [3] |
| SORL1 | rs1131497 | PET | Apostolova et al., (2018) [3] |
| CLU | rs9331949 | PET | Apostolova et al., (2018) [3] |
| DSG2 | rs8093731 | PET | Apostolova et al., (2018) [3] |
| EPHA1 | rs11767557 | PET | Hughes et al., (2014) [4] |
| CR1 | rs646817 | CSF Aβ | Brouwers et al., (2012) [24] |
| CR1 | rs1746659 | CSF Aβ | Brouwers et al., (2012) [24] |
| CR1 | rs12034383 | CSF Aβ | Brouwers et al., (2012) [24] |
| CR1 | rs11803956 | CSF Aβ | Brouwers et al., (2012) [24] |
| CR1 | rs3818361 | PET | Thambisetty et al., (2013) [25] |
| CYP2C19 | rs4388808 | PET, CSF Aβ and postmortem Aβ | Benedet et al., (2018) [26] |
| IAPP | rs73069071 | PET | Roostaei et al., (2017) [27] |
| BCHE | rs509208 | PET | Ramanan et al., (2014) [28] |
| PVRL2 | rs6857 | PET | Ramanan et al., (2014) [28] |
| SORL1 | rs1699102 | CSF Aβ | Guo et al., (2012) [29] |
| SORL1 | rs3824968 | CSF Aβ | Guo et al., (2012) [29] |
| USF1 | rs10908821 | Postmortem NP | Isotalo et al., (2012) [30] |
| USF1 | rs2774276 | Postmortem NP | Isotalo et al., (2012) [30] |
| USF1 | rs2516839 | Postmortem NP | Isotalo et al., (2012) [30] |
| IL1RAP | rs12053868 | PET | Ramanan et al., (2015) [31] |
| A2M | rs226379 | CSF Aβ | Millard et al., (2014) [32] |
| A2M | rs1805667 | CSF Aβ | Millard et al., (2014) [32] |
| RAP2B,C3orf79 | rs4680057 | PET | Yan et al., (2018) [33] |
| CD33 | rs3865444 | PET | Bradshaw et al., (2013) [34] |
| APOC1 | rs439401* | CSF Aβ | Souza et al., (2016) [35] |
| TOMM40 | rs2075650* | CSF Aβ | Souza et al., (2016) [35] |
|  |  | CSF Aβ | Han et al., (2010) [15] |
|  |  | CSF Aβ | Kim et al., (2010) [36] |
| TOMM40 | rs157580* | CSF Aβ | Kim et al., (2010) [36] |
| PLD3 | rs11667768 ** | CSF Aβ | Wang et al., (2015) [37] |
| SORL1 | rs2070045 ** | CSF Aβ | Guo et al., (2012) [29] |
| FERMT2 | rs17125944 ** | PET | Apostolova et al., (2018) [3] |
| GLIS1 | rs185031519 *** | CSF Aβ | Deming et al., (2017) [38] |
| SERPINB1 | rs316341 *** | CSF Aβ | Deming et al., (2017) [38] |
| SPATA8 - RN7SKP181 | rs1503351 *** | CSF Aβ | Li et al., (2015) [18] |
| GSK3B | rs1154597 *** | CSF Aβ | Kettunen et al., (2015) [20] |
| SLC35F4 | rs187911 *** | Postmortem NP | Shulman et al., (2013) [5] |
| ADCY8,EFR3A | rs13260032 *** | PET | Yan et al., (2018) [33] |

**Table S1** List of 78 SNPs associated with amyloid pathology measured by positron emission tomography (PET) scan, cerebrospinal fluid (CSF) amyloid or post-mortem measurement from systematic review. NP, neuritic plaque. * three SNPs were excluded because they were within 1Mb of *APOE*; ** three SNPs were excluded because they were also associated with total tau or phosphorylated tau, *** six SNPs were excluded because their effect alleles were not found in SNP genotyping data. After quality control, sixty-six SNPs were left and used for final Mendelian Randomization analysis.

| **Genes** | **SNPs** | **T-tau measurement** | **Reference** |
| --- | --- | --- | --- |
| MAPT | rs2471738 | CSF | Babic Leko et al., (2018) [39] |
| PBMC | rs2927438 | CSF | Rao et al., (2018) [40] |
| SRRM4 | rs10775009 | CSF | Chung et al., (2018) [41] |
| PLD4 and C14orf79 | rs2819438 | CSF | Chung et al., (2018) [41] |
| SORL1 | rs11218343 | CSF | Louwersheimer et al., (2016) [42] |
| GEMC1 and OSTN | rs9877502 | CSF | Cruchaga et al., (2013) [43] |
| GLIS3 | rs514716 | CSF | Cruchaga et al., (2013) [43] |
| SORL1 | rs661057 | CSF | Guo et al., (2012) [29] |
| CDH4 | rs4925189 | CSF | Han et al., (2010) [15] |
| FLJ21511 | rs2768975 | CSF | Han et al., (2010) [15] |
| LOC105370020 | rs1997111 | CSF | Han et al., (2010) [15] |
| / | rs6850199 | CSF | Han et al., (2010) [15] |
| GMNC | rs35055419 | CSF | Deming et al., (2017) [38] |
| GSK3B | rs334558 | CSF | Kettunen et al., (2015) [20] |
| PPP3R1 | rs1868402 | CSF | Elias-Sonnenschein et al., (2013) [1] |
| MAPT | rs2435211 | CSF | Elias-Sonnenschein et al., (2013) [1] |
| CFB | rs4151667 | CSF | Daborg et al., (2013) [44] |
| C2 | rs9332739 | CSF | Daborg et al., (2013) [44] |
| PLD3 | rs11667768 * | CSF | Tan et al., (2018) [45] |
| FERMT2 | rs17125944 * | CSF | Martiskainen et al., (2015) [6] |
| SORL1 | rs2070045 * | CSF | Louwersheimer et al., (2015) [46] |
| EPC2 | rs1374441 ** | CSF | Kim et al., (2011) [36] |
| EPC2 | rs2121433 ** | CSF | Kim et al., (2011) [36] |
| MAPT | rs1467967 ** | CSF | Babic Leko et al., (2018) [39] |

**Table S2** List of 24 SNPs associated with cerebrospinal fluid (CSF) total tau (t-tau) measurement from systematic review. * three SNPs were excluded because they were also associated with amyloid pathology, ** three SNPs were excluded because their effect alleles were not found in SNP genotyping data. After quality control, eighteen SNPs were left and used for final Mendelian Randomization analysis.

| Uniprot | Protein Name |
| --- | --- |
| Q9NX18 | Succinate dehydrogenase assembly factor 2, mitochondrial |
| Q9NP66 | High mobility group protein 20A |
| Q6IQ23 | Pleckstrin homology domain-containing family A member 7 |
| P24298 | Alanine aminotransferase 1 |
| Q96DZ1* | Endoplasmic reticulum lectin 1 |

**Table S3** Five proteins reached false discovery rate (FDR) correction (*q* < 0.1) from partial correlation in group one. * Q96DZ1 was selected in 44 protein panel.

| Uniprot | Protein Name |
| --- | --- |
| Q8NEW7 | Transmembrane inner ear expressed protein |
| O75347 | Tubulin-specific chaperone A |
| Q9UBQ0 | Vacuolar protein sorting-associated protein 29 |
| Q9NY15 | Stabilin-1 |
| P23435 | Cerebellin-1 |
| Q07954 | Prolow-density lipoprotein receptor-related protein 1 |
| Q15116 | Programmed cell death protein 1 |
| Q9BQ69 | O-acetyl-ADP-ribose deacetylase MACROD1 |
| Q9NPH3 | Interleukin-1 Receptor accessory protein |
| Q9HB29 | Interleukin-1 receptor-like 2 |
| P19419 | ETS domain-containing protein Elk-1 |
| P01374 | Lymphotoxin-alpha |
| P41222 | Prostaglandin-H2 D-isomerase |
| Q03154 | Aminoacylase-1 |
| O00764 | Pyridoxal kinase |
| Q96A25 | Transmembrane protein 106A |
| Q8WWA1 | Transmembrane protein 40 |
| P23381 | Tryptophan--tRNA ligase, cytoplasmic |
| Q9BSI4 | TERF1-interacting nuclear factor 2 |
| P09104 | Gamma-enolase |
| O94929 | Actin-binding LIM protein 3 |
| P52797 | Ephrin-A3 |
| P53365 | Arfaptin-2 |
| Q96DZ1* | Endoplasmic reticulum lectin 1 |
| P52758 | Ribonuclease UK114 |
| O15335 | Chondroadherin |
| A6NKW6 | Membrane protein FAM159B |
| Q9BQT9 | Calsyntenin-3 |
| Q96PJ5 | Fc receptor-like protein 4 |
| Q16623 | Syntaxin-1A |
| Q5T7V8 | RAB6-interacting golgin |
| P26572 | Alpha-1,3-mannosyl-glycoprotein 2-beta-N-acetylglucosaminyltransferase |
| Q8WWZ8 | Oncoprotein-induced transcript 3 protein |
| P26951 | Interleukin-3 receptor subunit alpha |
| P28906 | Hematopoietic progenitor cell antigen CD34 |
| P60985 | Keratinocyte differentiation-associated protein |
| Q99983 | Osteomodulin |
| Q8N4E7 | Ferritin, mitochondrial |
| Q9NR71 | Neutral ceramidase |
| Q5VU65 | Nuclear pore membrane glycoprotein 210-like |
| P21128 | Poly(U)-specific endoribonuclease |
| P04440 | HLA class II histocompatibility antigen, DP beta 1 chain |
| P02747 | Complement C1q subcomponent subunit C |
| Q6X784 | Zona pellucida-binding protein 2 |

**Table S4** 44 proteins selected from machine learning to differentiate between high and low Aβ in group one, the rank was based on their contribution to the classification from the largest to smallest. * Q96DZ1 reached false discovery rate (FDR) correction (*q* < 0.1) from partial correlation.

| **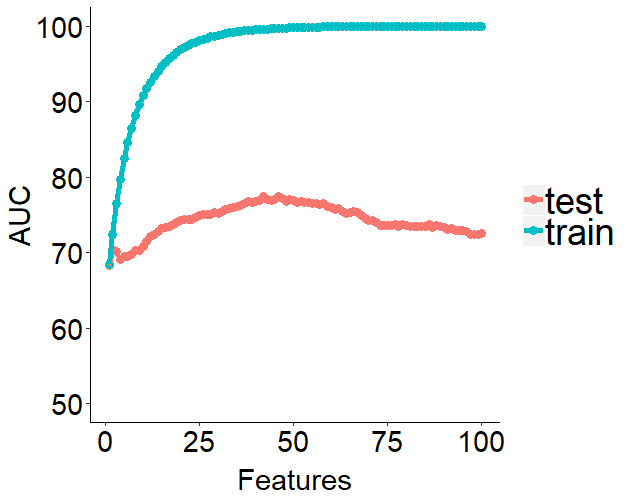** |
| --- |

**Figure S1**. The AUC in training and testing sets for different number of input features in differentiating between high and low Aβ in group one, a panel of 46 features achieved the highest predictive value (Area Under Curve (AUC) of 0.78).

| Outcomes | Sample size | AUC |
| --- | --- | --- |
| AD vs HC | 184 AD / 311 HC | 0.65 |
| AD vs MCI | 184 AD / 386 MCI | 0.63 |
| T-tau | 791 | 0.66 |
| P-Tau | 787 | 0.65 |
| MCI conversion | 319 | 0.62 |
| MMSE baseline | 786 | 0.60 |
| Hippocampal volume | 576 | 0.63 |
| NFL | 570 | 0.54 |
| Neurogranin | 534 | 0.54 |
| YKL-40 | 576 | 0.53 |

**Table S5** Area under the curve (AUC) of the 46 signatures to differentiate AD from HC, AD from MCI, status of T-tau, P-tau, MCI conversion, baseline MMSE score, hippocampal volume, and other three CSF biomarkers including NFL, neurogranin and YKL-40. HC, healthy control; MCI, mild cognitive impairment; T-tau, total tau; P-tau, phosphorylated tau; MMSE, mini mental state examination; NFL, neurofilament light chain.

| 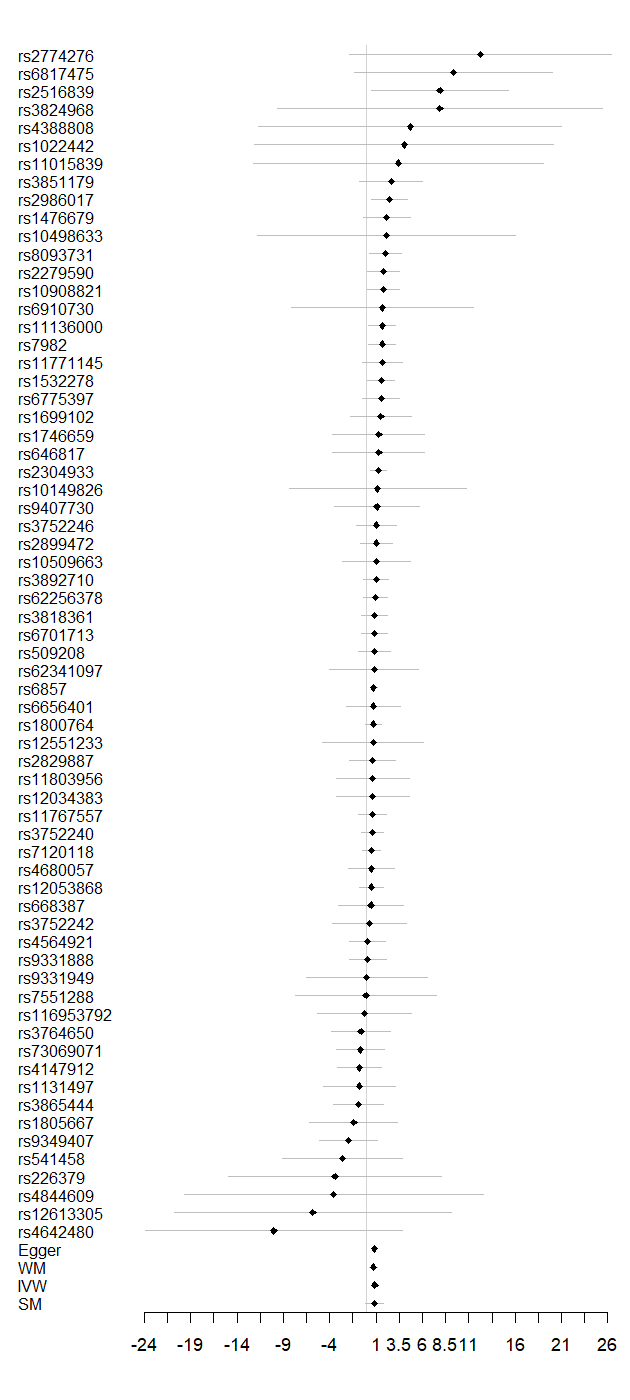 | 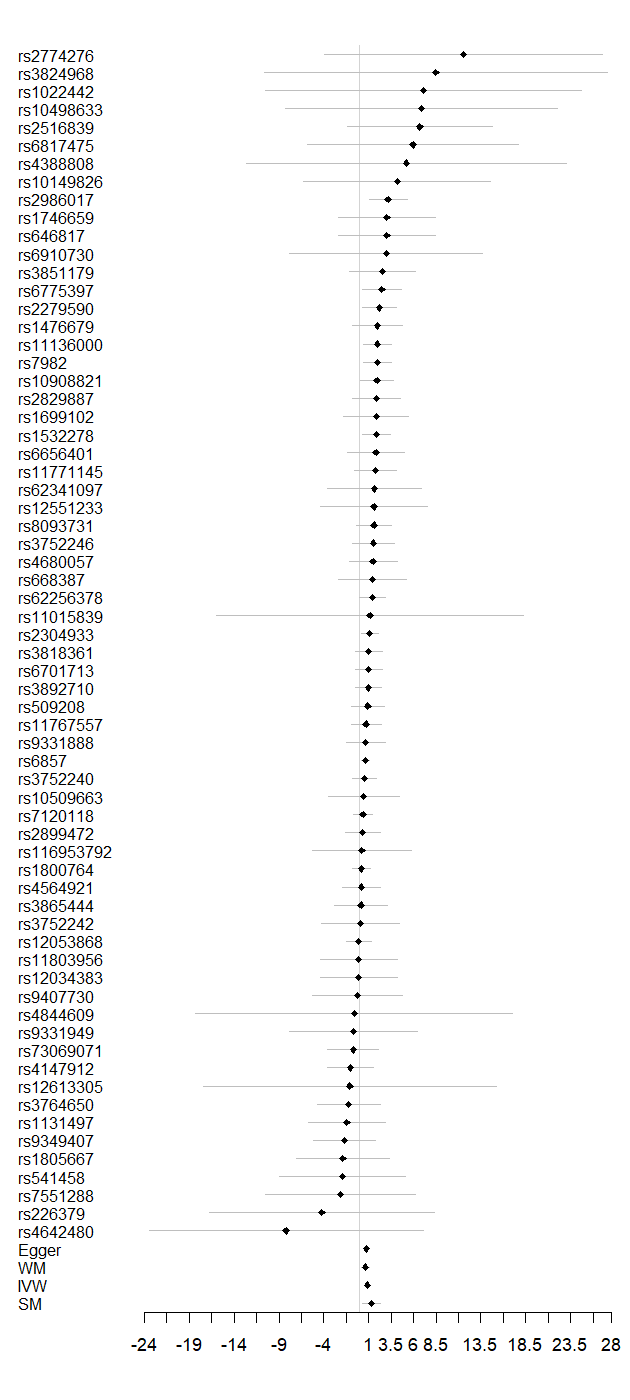 |
| --- | --- |
| (a) | (b) |

**Figure S2** Forest plot of MR estimates the effects of amyloid on t-tau (a) and p-tau (b). The estimated effect size of MR Egger method, expressed as a β, was as follows: t-tau (β = 0.85, se = 0.15, 95% CI [0.54, 1.15], p < 0.001), and p-tau (β = 0.73, se = 0.17, 95% CI [0.39, 1.06], *p* < 0.001); the estimated effect size of weighted median (WM) method for t-tau (β = 0.73, se = 0.22, 95% CI [0.31, 1.16], *p* < 0.001) and for p-tau (β = 0.62, se = 0.23, 95% CI [0.17, 1.06], *p* = 0.006); the estimated effect size of inverse-variance weighted (IVW) method for t-tau (β = 0.86, se = 0.11, 95% CI [0.65, 1.08], *p* < 0.001) and for p-tau (β = 0.86, se = 0.12, 95% CI [0.62, 1.10], *p* < 0.001); the estimated effect size of simple median (SM) method for t-tau (β = 0.81, se = 0.50, 95% CI [-0.17, 1.80], *p* = 0.11) and for p-tau (β = 1.31, se = 0.52, 95% CI [0.29, 2.33], *p* = 0.01).

| **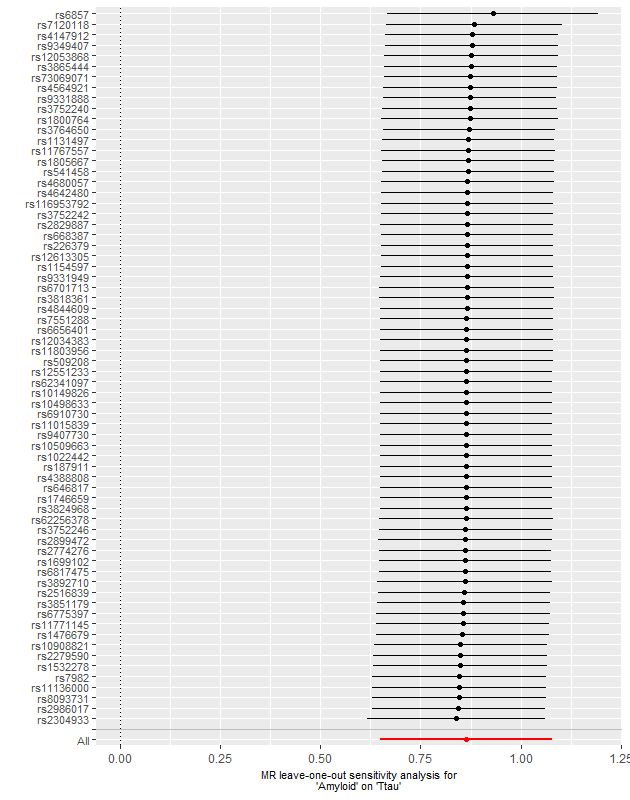** | **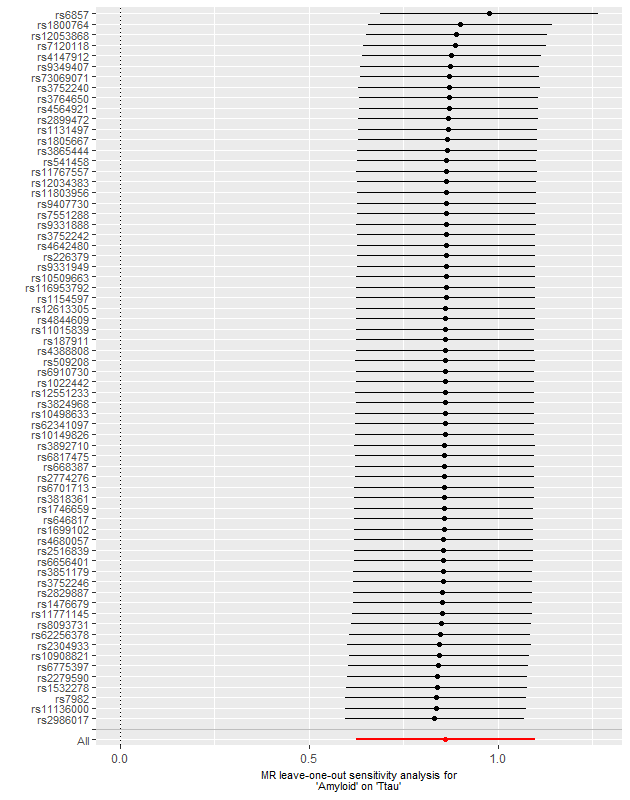** |
| --- | --- |
| (a) | (b) |

**Figure S3** Leave-one-out Mendelian Randomization estimates the association of amyloid and total tau (a) as well as amyloid and phosphorylated tau (b) by sequentially removing each single-nucleotide polymorphism (SNP) from the analysis. No single SNP drove the majority of the association signal between amyloid and tau or amyloid and p-tau.

|  | Methods | β | se | 95% CI | *P* value |
| --- | --- | --- | --- | --- | --- |
| Amyloid SNPs with NFL | MR Egger | 0.03 | 0.05 | -0.07 to 0.12 | 0.59 |
|  | Weighted median | 0.04 | 0.05 | -0.06 to 0.13 | 0.44 |
|  | IVW | 0.03 | 0.03 | -0.03 to 0.10 | 0.29 |
|  | Simple median | 0.13 | 0.12 | -0.11 to 0.36 | 0.29 |
| Amyloid SNPs with Neurogranin | MR Egger | -0.45 | 0.21 | -0.86 to 0.05 | 0.13 |
|  | Weighted median | -0.37 | 0.24 | -0.83 to -0.09 | 0.12 |
|  | IVW | -0.32 | 0.14 | -0.61 to 0.04 | 0.13 |
|  | Simple median | -0.08 | 0.48 | -1.02 to 0.87 | 0.87 |
| Amyloid SNPs with YKL-40 | MR Egger | -0.08 | 0.12 | -0.33 to 0.16 | 0.50 |
|  | Weighted median | -0.01 | 0.14 | -0.28 to 0.26 | 0.96 |
|  | IVW | -0.14 | 0.09 | -0.31 to 0.03 | 0.11 |
|  | Simple median | -0.05 | 0.36 | -0.75 to 0.66 | 0.90 |
| Tau SNPs with NFL | MR Egger | -0.09 | 0.11 | -0.30 to 0.12 | 0.41 |
|  | Weighted median | -0.08 | 0.08 | -0.24 to 0.07 | 0.31 |
|  | IVW | -0.10 | 0.06 | -0.22 to 0.02 | 0.10 |
|  | Simple median | -0.10 | 0.10 | -0.29 to 0.09 | 0.29 |
| Tau SNPs with Neurogranin | MR Egger | -0.13 | 0.17 | -0.46 to -0.02 | **0.043** |
|  | Weighted median | -0.32 | 0.12 | -0.55 to -0.09 | **0.006** |
|  | IVW | -0.24 | 0.08 | -0.40 to -0.08 | **0.004** |
|  | Simple median | -0.34 | 0.13 | -0.60 to -0.09 | **0.009** |
| Tau SNPs with YKL-40 | MR Egger | -0.35 | 0.17 | -0.68 to -0.03 | **0.03** |
|  | Weighted median | -0.38 | 0.13 | -0.63 to -0.12 | **0.004** |
|  | IVW | -0.34 | 0.08 | -0.50 to -0.18 | **0.000** |
|  | Simple median | -0.31 | 0.13 | -0.57 to -0.05 | **0.02** |

**Table S6** Mendelian Randomization estimates of the causal effect of both amyloid and tau on neurofilament light chain (NFL), Neurogranin and YKL-40; β, beta coefficient; CI, confidence interval; IVW, Inverse-variance weighted.

**References**

1. Elias-Sonnenschein, L.S., et al., Genetic loci associated with Alzheimer's disease and cerebrospinal fluid biomarkers in a Finnish case-control cohort. PLoS One, 2013. 8(4): p. e59676.

2. Tan, L., et al., Effect of CLU genetic variants on cerebrospinal fluid and neuroimaging markers in healthy, mild cognitive impairment and Alzheimer's disease cohorts. Sci Rep, 2016. 6: p. 26027.

3. Apostolova, L.G., et al., Associations of the Top 20 Alzheimer Disease Risk Variants With Brain Amyloidosis. JAMA Neurol, 2018. 75(3): p. 328-341.

4. Hughes, T.M., et al., Markers of cholesterol transport are associated with amyloid deposition in the brain. Neurobiol Aging, 2014. 35(4): p. 802-7.

5. Shulman, J.M., et al., Genetic susceptibility for Alzheimer disease neuritic plaque pathology. JAMA Neurol, 2013. 70(9): p. 1150-7.

6. Martiskainen, H., et al., Effects of Alzheimer's disease-associated risk loci on cerebrospinal fluid biomarkers and disease progression: a polygenic risk score approach. J Alzheimers Dis, 2015. 43(2): p. 565-73.

7. Koppel, J., et al., CALHM1 P86L polymorphism modulates CSF Abeta levels in cognitively healthy individuals at risk for Alzheimer's disease. Mol Med, 2011. 17(9-10): p. 974-9.

8. Kauwe, J.S., et al., Validating predicted biological effects of Alzheimer's disease associated SNPs using CSF biomarker levels. J Alzheimers Dis, 2010. 21(3): p. 833-42.

9. Zhu, X.C., et al., Effect of CR1 Genetic Variants on Cerebrospinal Fluid and Neuroimaging Biomarkers in Healthy, Mild Cognitive Impairment and Alzheimer's Disease Cohorts. Mol Neurobiol, 2017. 54(1): p. 551-562.

10. Zhao, Q.F., et al., ABCA7 Genotypes Confer Alzheimer's Disease Risk by Modulating Amyloid-beta Pathology. J Alzheimers Dis, 2016. 52(2): p. 693-703.

11. Kok, E.H., et al., CLU, CR1 and PICALM genes associate with Alzheimer's-related senile plaques. Alzheimers Res Ther, 2011. 3(2): p. 12.

12. Schjeide, B.M., et al., The role of clusterin, complement receptor 1, and phosphatidylinositol binding clathrin assembly protein in Alzheimer disease risk and cerebrospinal fluid biomarker levels. Arch Gen Psychiatry, 2011. 68(2): p. 207-13.

13. Chibnik, L.B., et al., CR1 is associated with amyloid plaque burden and age-related cognitive decline. Ann Neurol, 2011. 69(3): p. 560-9.

14. Alexopoulos, P., et al., Impact of SORL1 single nucleotide polymorphisms on Alzheimer's disease cerebrospinal fluid markers. Dement Geriatr Cogn Disord, 2011. 32(3): p. 164-70.

15. Han, M.R., G.D. Schellenberg, and L.S. Wang, Genome-wide association reveals genetic effects on human Abeta42 and tau protein levels in cerebrospinal fluids: a case control study. BMC Neurol, 2010. 10: p. 90.

16. Kauwe, J.S., et al., Alzheimer's disease risk variants show association with cerebrospinal fluid amyloid beta. Neurogenetics, 2009. 10(1): p. 13-7.

17. Swaminathan, S., et al., Amyloid pathway-based candidate gene analysis of [(11)C]PiB-PET in the Alzheimer's Disease Neuroimaging Initiative (ADNI) cohort. Brain Imaging Behav, 2012. 6(1): p. 1-15.

18. Li, Q.S., et al., Variations in the FRA10AC1 Fragile Site and 15q21 Are Associated with Cerebrospinal Fluid Abeta1-42 Level. PLoS One, 2015. 10(8): p. e0134000.

19. Ramirez, A., et al., SUCLG2 identified as both a determinator of CSF Abeta1-42 levels and an attenuator of cognitive decline in Alzheimer's disease. Hum Mol Genet, 2014. 23(24): p. 6644-58.

20. Kettunen, P., et al., Genetic variants of GSK3B are associated with biomarkers for Alzheimer's disease and cognitive function. J Alzheimers Dis, 2015. 44(4): p. 1313-22.

21. Natunen, T., et al., Effects of NR1H3 genetic variation on the expression of liver X receptor alpha and the progression of Alzheimer's disease. PLoS One, 2013. 8(11): p. e80700.

22. Beecham, G.W., et al., Genome-wide association meta-analysis of neuropathologic features of Alzheimer's disease and related dementias. PLoS Genet, 2014. 10(9): p. e1004606.

23. Replogle, J.M., et al., A TREM1 variant alters the accumulation of Alzheimer-related amyloid pathology. Ann Neurol, 2015. 77(3): p. 469-77.

24. Brouwers, N., et al., Alzheimer risk associated with a copy number variation in the complement receptor 1 increasing C3b/C4b binding sites. Mol Psychiatry, 2012. 17(2): p. 223-33.

25. Thambisetty, M., et al., Effect of complement CR1 on brain amyloid burden during aging and its modification by APOE genotype. Biol Psychiatry, 2013. 73(5): p. 422-8.

26. Benedet, A.L., et al., CYP2C19 variant mitigates Alzheimer disease pathophysiology in vivo and postmortem. Neurology Genetics, 2018. 4(1): p. e216.

27. Roostaei, T., et al., Genome-wide interaction study of brain beta-amyloid burden and cognitive impairment in Alzheimer's disease. Mol Psychiatry, 2017. 22(2): p. 287-295.

28. Ramanan, V.K., et al., APOE and BCHE as modulators of cerebral amyloid deposition: a florbetapir PET genome-wide association study. Mol Psychiatry, 2014. 19(3): p. 351-7.

29. Guo, L.H., et al., SORL1 genetic variants and cerebrospinal fluid biomarkers of Alzheimer's disease. Eur Arch Psychiatry Clin Neurosci, 2012. 262(6): p. 529-34.

30. Isotalo, K., et al., Upstream transcription factor 1 (USF1) polymorphisms associate with Alzheimer's disease-related neuropathological lesions: Tampere Autopsy Study. Brain Pathol, 2012. 22(6): p. 765-75.

31. Ramanan, V.K., et al., GWAS of longitudinal amyloid accumulation on 18F-florbetapir PET in Alzheimer's disease implicates microglial activation gene IL1RAP. Brain, 2015. 138(Pt 10): p. 3076-88.

32. Millard, S.P., et al., Association of cerebrospinal fluid Abeta42 with A2M gene in cognitively normal subjects. Neurobiol Aging, 2014. 35(2): p. 357-64.

33. Yan, Q., et al., Genome-wide association study of brain amyloid deposition as measured by Pittsburgh Compound-B (PiB)-PET imaging. Mol Psychiatry, 2018.

34. Bradshaw, E.M., et al., CD33 Alzheimer's disease locus: altered monocyte function and amyloid biology. Nat Neurosci, 2013. 16(7): p. 848-50.

35. Souza, M.B., et al., Combined Genome-Wide CSF Abeta-42's Associations and Simple Network Properties Highlight New Risk Factors for Alzheimer's Disease. J Mol Neurosci, 2016. 58(1): p. 120-8.

36. Kim, S., et al., Genome-wide association study of CSF biomarkers Abeta1-42, t-tau, and p-tau181p in the ADNI cohort. Neurology, 2011. 76(1): p. 69-79.

37. Wang, C., et al., Common Variants in PLD3 and Correlation to Amyloid-Related Phenotypes in Alzheimer's Disease. J Alzheimers Dis, 2015. 46(2): p. 491-5.

38. Deming, Y., et al., Genome-wide association study identifies four novel loci associated with Alzheimer's endophenotypes and disease modifiers. Acta Neuropathol, 2017. 133(5): p. 839-856.

39. Babic Leko, M., et al., Association of MAPT haplotype-tagging polymorphisms with cerebrospinal fluid biomarkers of Alzheimer's disease: A preliminary study in a Croatian cohort. Brain Behav, 2018: p. e01128.

40. Rao, S., et al., An APOE-independent cis-eSNP on chromosome 19q13.32 influences tau levels and late-onset Alzheimer's disease risk. Neurobiol Aging, 2018. 66: p. 178.e1-178.e8.

41. Chung, J., et al., Genome-wide association study of Alzheimer's disease endophenotypes at prediagnosis stages. Alzheimers Dement, 2018. 14(5): p. 623-633.

42. Louwersheimer, E., et al., Alzheimer's disease risk variants modulate endophenotypes in mild cognitive impairment. Alzheimers Dement, 2016. 12(8): p. 872-81.

43. Cruchaga, C., et al., GWAS of cerebrospinal fluid tau levels identifies risk variants for Alzheimer's disease. Neuron, 2013. 78(2): p. 256-68.

44. Daborg, J., et al., Complement gene single nucleotide polymorphisms and biomarker endophenotypes of Alzheimer's disease. J Alzheimers Dis, 2013. 35(1): p. 51-7.

45. Tan, M.S., et al., Common Variant in PLD3 Influencing Cerebrospinal Fluid Total Tau Levels and Hippocampal Volumes in Mild Cognitive Impairment Patients from the ADNI Cohort. J Alzheimers Dis, 2018. 65(3): p. 871-876.

46. Louwersheimer, E., et al., Influence of genetic variants in SORL1 gene on the manifestation of Alzheimer's disease. Neurobiol Aging, 2015. 36(3): p. 1605.e13-20.
